# Leveraging Dynamic Electrostatic and Hydrophobic Interactions for Biomedical Hydrogels

**DOI:** 10.1021/acsmacrolett.6c00090

**Published:** 2026-03-31

**Authors:** Olivia F. Dingus, Melissa A. Grunlan

**Affiliations:** † Department of Biomedical Engineering, 2655 Texas A&M University, College Station, Texas 77843-3003, United States; ‡ Department of Materials Science & Engineering, Texas A&M University, College Station, Texas 77843-3003, United States; § Department of Chemistry, Texas A&M University, College Station, Texas 77843-3003, United States

## Abstract

The reversibility of dynamic cross-links affords hydrogel-enhanced responsiveness to external stimuli, making them useful in a variety of biomedical applications. While numerous types of dynamic interactions exist, electrostatic and hydrophobic interactions are notably potent. Herein, recent reports are highlighted that demonstrate the utility of these interactions to form hydrogels with notable properties such as stimuli-responsiveness, adhesivity, injectability, self-healing, and toughness. While most often utilized independently, emerging reports show that hydrogels can be formed with a synergistic combination of electrostatic and hydrophobic interactions. The applications of such dynamic hydrogels are broad but include drug delivery, wound healing, cartilage regeneration, and sensing. The highlighted reports underscore the diversity of approaches that can be employed to impart and tailor electrostatic and hydrophobic interactions to hydrogels, including polymer chemistry and architecture, as well as network design that may also include covalent cross-linking.

## Graphical abstract

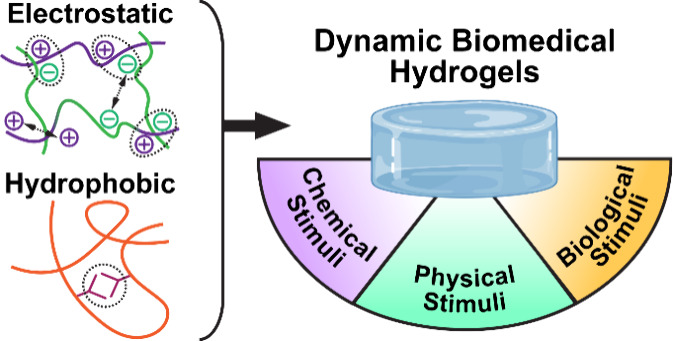

## 1 Introduction

Hydrogels are water-swollen, three-dimensional polymer networks.^1^ Owing to abundant water content, hydrogels have been utilized in an array of biomedical applications,^2–4^ including drug carriers,^5–8^ wound care,^9–12^ tissue engineering,^13–15^ and sensing.^16–18^ The nature of the chemical and/or physical cross-links used to form hydrogels is instrumental to the resulting properties. Chemically cross-linked polymer networks may be produced by covalent, irreversible bonding with a variety of methods (e.g., chain growth, step growth, enzymatic reactions, irradiation, and “click” chemistry).^19–21^ As such, with the permanent nature of such cross-links, the resulting hydrogels are somewhat static and have limited capacity to temporally respond to external stimuli and to achieve biological mimicry.^22–24^ This has prompted interest in dynamic hydrogels (i.e., supramolecular hydrogels) formed with dynamic cross-links that may be covalent or physical. Dynamic *covalent* cross-linking is afforded by reversible, covalent interactions (e.g., reversible exchange reactions, reversible addition/condensation reactions, coordinate interactions, and enzymatic/mechanical covalent reactions).^25^ In contrast, dynamic *physical* cross-links are produced from reversible, noncovalent interactions (e.g., electrostatic interactions, hydrophobic interactions, hydrogen bonds, metal–ligand coordination, and host–guest interactions).^26,27^ Overall, dynamic cross-linking may provide hydrogels with responsiveness to a variety of external cues including chemical (e.g., pH, ions, and redox), physical (e.g., light, temperature, mechanical stress, and magnetic fields), or biological (e.g., cells, enzymes, and proteins).^28–34^ Dynamic cross-links also act as sacrificial bonds, affording dissipation of applied stress, resulting in hydrogels with adhesivity, injectability, self-healing, high extensibility, and/or tissue-like mechanical properties.^35–39^ Overall, the unique properties of hydrogel systems based on dynamic interactions have prompted their utility in a variety of biomedical applications, including controlled release, wound healing, regenerative engineering, tissue substitutes, and sensing.^40,41^

Hydrogel designs that leverage dynamic interactions hold tremendous potential to create a breadth of next-generation biomedical devices. This viewpoint highlights recent reports of hydrogels formed specifically via electrostatic interactions or hydrophobic interactions (Figure 1). Several reports demonstrate that these interactions may be synergistically combined within a hydrogel, as well. Imparted by their dynamic nature, hydrogels prepared with electrostatic or hydrophobic interactions display responsiveness to various stimuli, resulting in unprecedented and tunable functional properties useful in numerous biomedical applications.

**Figure 1:**
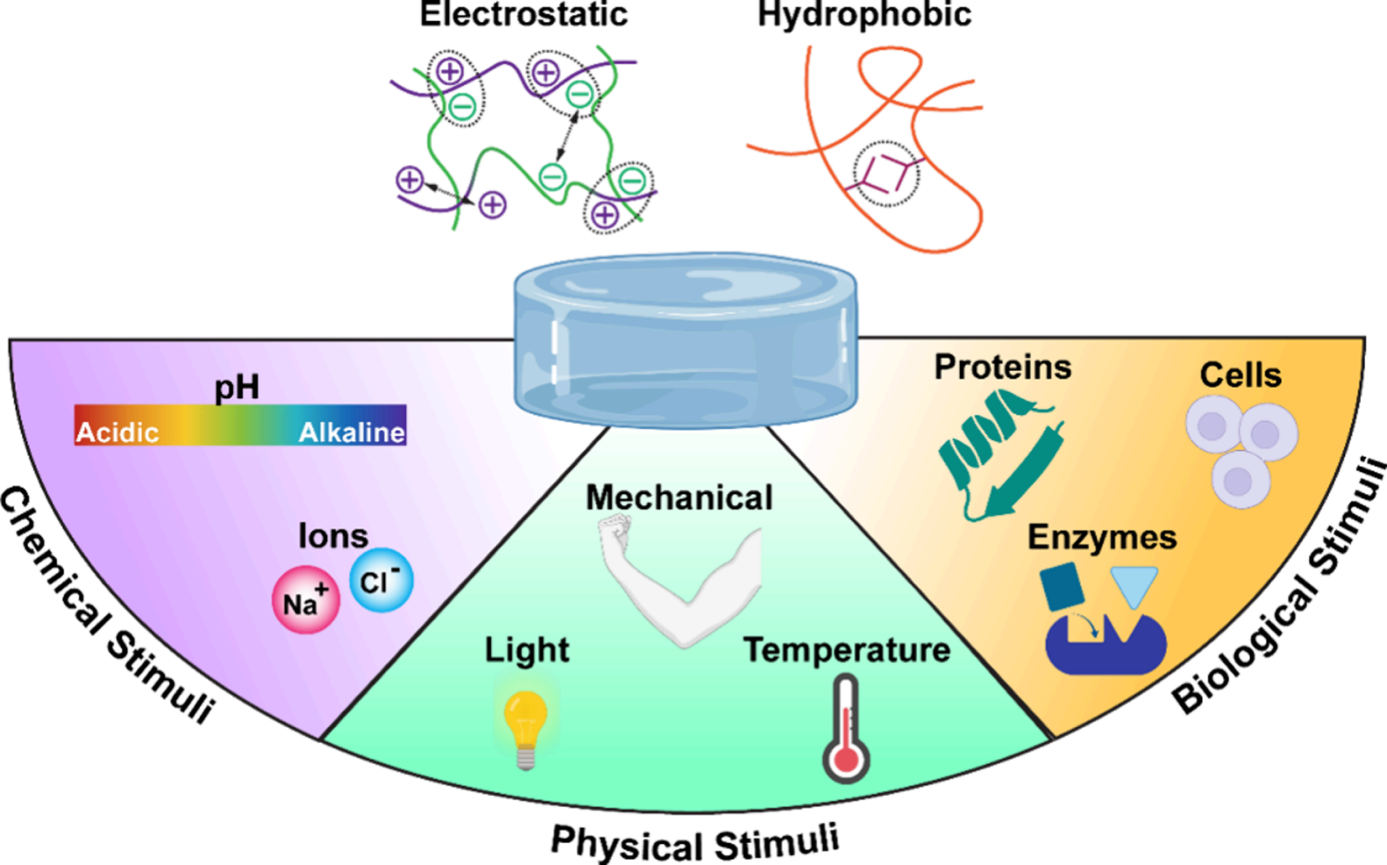
Hydrogels formed with dynamic electrostatic interactions and/or hydrophobic interactions are responsive to external stimuli such as chemical, physical, and biological stimuli and thereby produce functionality useful in a myriad of biomedical applications.

## 2 Electrostatic Interactions

Polyelectrolytes (PEs) are often used to form polyelectrolyte complexes (PECs)^42^ or polyampholyte (PA) hydrogels.^43^ PEs have the potential to display dynamic electrostatic interactions stemming from charged moieties (Figure 2). The International Union of Pure and Applied Chemistry (IUPAC) defines PEs as a “polymer composed of macromolecules in which a substantial portion of the constitutional units contains ionic or ionizable groups, or both”.^44^ These ionizable groups dissociate upon dissolving in water or other polar solvents, giving rise to a polyion (i.e., macro-ion) surrounded by mobile counterions.^45,46^ PEC hydrogels are formed by a combination of oppositely charged PEs (i.e., a negatively charged ‘polyanion’ and a positively charged ‘polycation’). In the case of zwitterionic PEs, both anionic and cationic charges are present.^47,48^ PAs specifically bear anionic and cationic charge on the separate repeat units while polybetaines have these charges on the same repeat unit.^49,50^ PA hydrogels are traditionally formed via copolymerization of oppositely charged monomers in ratios that may or may not maintain charge balance.^51,52^ PEs can also be classified as strong or weak PEs based on their ability to dissociate fully or partially, respectively, in water. Strong PEs thus maintain a constant charge regardless of pH, while the charge of weak PEs is highly influenced by pH.^53–55^ PEs imbue the resulting PEC and PA hydrogels with electrostatic interactions that may be attractive and/or repulsive and may occur within a single chain (″intrachain”) and between different chains ("interchain"). Magnification and tuning of these electrostatic interactions may be achieved using PEs of complex architectures (e.g., multiarm, multiblock, etc.) and network designs (e.g., multinetwork, interpenetrating networks [IPNs], and semi-IPNs). Beyond forming PEC and PAs exclusively via electrostatic interactions, covalent cross-links may also be introduced to achieve desired properties. To form hydrogels these PEC and PA interactions may use a variety of ionizable polymers including anionic, cationic, and zwitterionic PEs (Figure 3).

**Figure 2:**
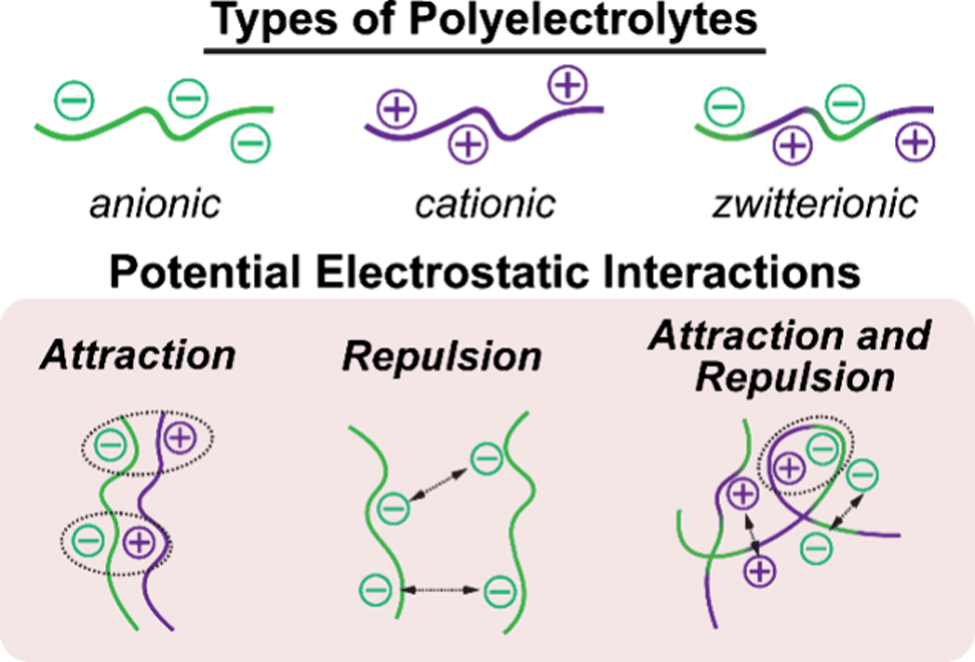
Schematic of polyelectrolytes (PEs) and possible electrostatic interactions within hydrogels.

**Figure 3:**
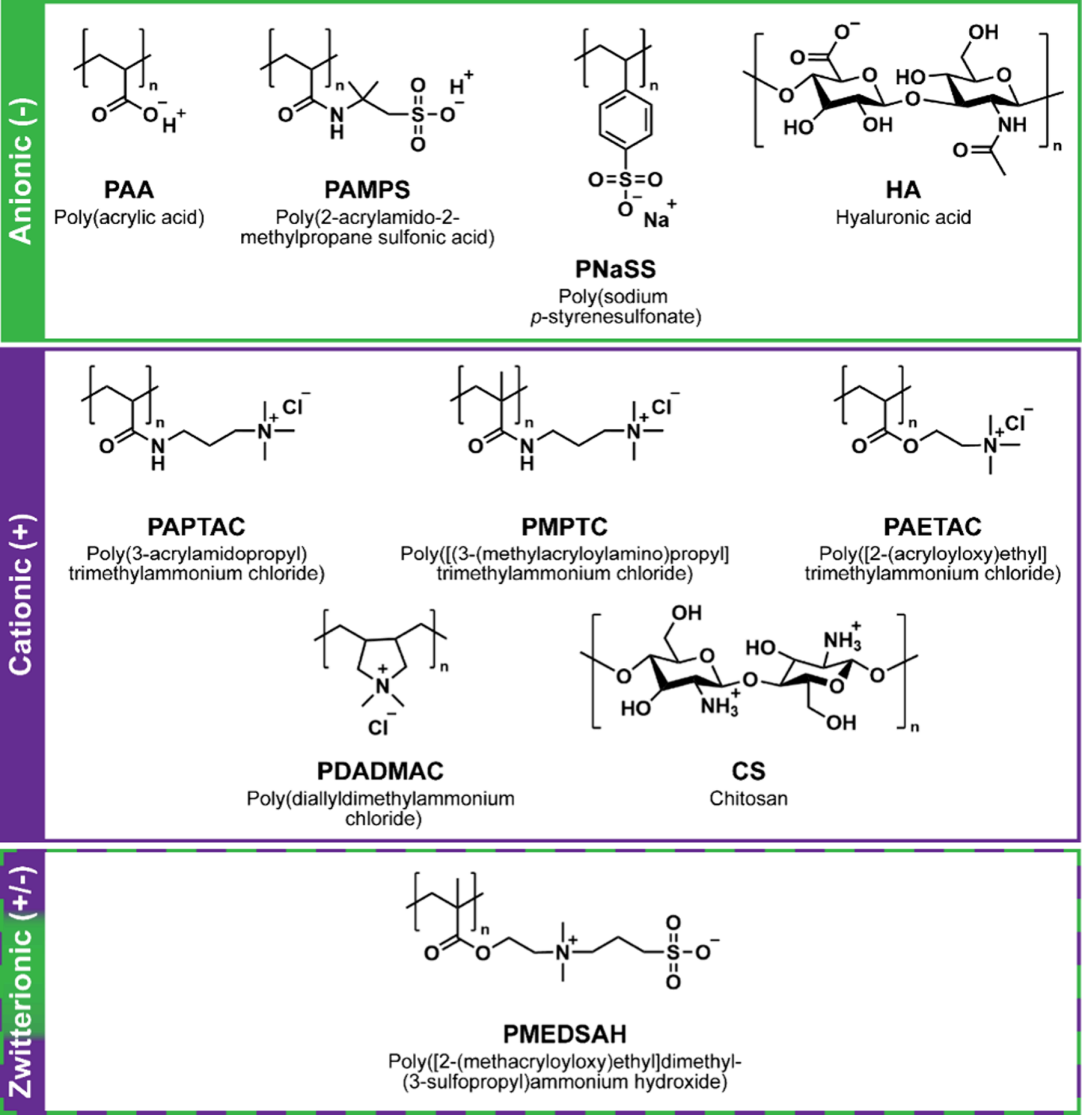
Commonly used polyelectrolytes (PEs) with electrostatic interactions for biomedical hydrogels.

Dynamic electrostatic interactions stemming from PEs have been utilized to create PEC and PA hydrogels with a variety of functional properties (Table 1). As noted above, electrostatic interactions may exclusively serve as cross-links to form the hydrogel. For instance, Nguyen et al. combined oppositely charged multiarm copolyelectrolytes, with anionic sulfonate and cationic quaternary ammonium groups, to form extracellular matrix (ECM) mimetic hydrogels with tunable stiffness for 3D cell culture.^56^ Abraham et al. combined low molecular weight cationic and anionic fluorenylmethyloxy-carbonyl-modified phenylalanine (Fmoc-Phe) to prepare a shear-thinning hydrogel with sustained release of oppositely charged cargo.^57^ In Kim et al., cationic chitosan and anionic succinoglycan formed hydrogels that exhibited antibacterial behavior and pH-dependent controlled release.^58^ Electrostatic interactions have also been used to achieve coacervation (i.e., liquid–liquid phase separation) wherein a homogeneous solution demixes into a dense and a dilute phase.^59^ For instance, in Wang et al., a PE coacervate hydrogel prepared from cationic polyamidoamine-epichlorohydrin (PAE) and anionic tannic acid displayed underwater adhesivity that was reusable due to self-healing.^60^

**Table 1:** Recent Studies of Hydrogels That Leverage Dynamic Electrostatic Interactions^a^

	study	composition	key findings
**polyelectrolyte complex (PEC) hydrogels**	Nguyen, 2022 ^56^	Multiarm block PEs: PEG–PMAETAC [+], PEG–PSPMA [−]	PEC hydrogels formed exhibited shear thinning, self-recovery, and cellular encapsulation.
	Abraham, 2020 ^57^	Supramolecular charged Fmoc-Phe: ammonium-terminated [+] or carboxylate-terminated [−]	PEC hydrogels produced sustained release of oppositely charged cargo, while similarly charged or neutral charged cargo led to burst release.
	Kim, 2023 ^58^	PEs: SG [−], CS [+]	PEC hydrogels displayed antibacterial behavior, as well as pH sensitive controlled release.
	Wang, 2021 ^60^	PE: PAE [+]	PE coacervate hydrogel demonstrated underwater adhesivity in a wide range of pHs and salt concentrations, with self-healing affording reusability.
		Anionic: tannic acid [−]	
**covalently cross-linked PEC hydrogels**	Lv, 2023 ^61^	PEs: EPL [+], O-HA [−]	Schiff-base covalently cross-linked PEC hydrogels exhibited injectability, self-healing, adhesivity, hemostasis, and antibacterial behavior.
		Fe^3+^ [+]	
	Li, 2022 ^62^	Triblock PEs: PAGE–PEO-PAGE (functionalized by guanidium [+], ammonium [+], or sulfonate [−])	PEC-IPN hydrogels exhibited improved mechanical strength and stability, including salt environments, versus equivalent PEC hydrogels.
	Lu, 2021 ^63^	PE: P(DOPA-MPTC) [±]	PEC-IPN hydrogels exhibited tissue-like strength, adhesivity to tissue, and hemostatic capability.
		allyl-functionalized cellulose	
	Chen, 2023 ^64^	PEs: HACC [+], HA [−]	Covalently cross-linked PEC hydrogels exhibited high elasticity, tensile strength, and antibacterial behavior.
	Wang, 2020 ^65^	Monomers: AMPS [−], AETAC [+], MEDSAH [±]	Covalently cross-linked PEC adhesive, antibacterial hydrogels with high strain (∼900%) capacity.
	Iudin, 2025 ^66^	PEs: PDADMAC [+], HAMA [−]	Covalently cross-linked, 3D-printed PEC hydrogels exhibited postprint shrinking, with volume reduced by 9X and a resolution as low as 42 ± 6 μm.
**covalently cross-linked polyampholyte (PA) hydrogels**	Zhang, 2021 ^67^	Monomers: MPTC [+], DMAEMA [+], NaSS [−]	Covalently cross-linked PA hydrogels exhibited rapid self-healing and electroconductivity behavior.
	Shao, 2021 ^68^	Monomers: NaSS [−], DMAEA-Q [+]	Covalently cross-linked PA hydrogels exhibited shape recovery in salt solutions.
	Martinez, 2025 ^69^	PEs: PDADMAC [+]	Covalently cross-linked semi-IPN PA hydrogel exhibited electrostatic adhesion to an anionic, antibiofouling DN hydrogel rod to seal the cavity for retention of small molecules (e.g., optical glucose sensing assay).
		Momoners: NaSS [−], AETAC [+]	

a Abbreviations: Polyelectrolyte [PE], poly­(ethylene glycol)-*block*-poly­(2-[(methacryloyloxy)­ethyl]­trimethylammonium chloride solution) [PEG–PMAETAC], poly­(ethylene glycol)-*block*-poly­(3-sulfopropyl methacrylate potassium salt) [PEG–PSPMA], fluorenylmethyl-oxycarbonyl-modified phenylalanine [Fmoc-Phe], succinoglycan [SG], chitosan [CS], polyamidoamine-epichlorohydrin [PAE], ε-poly-l-lysine [EPL], oxidized hyaluronic acid [O-HA], poly­(allyl glycidyl)-*block*-poly­(ethylene glycol)- *block*-poly­(allyl glycidyl ether) [PAGE–PEO–PAGE], polyelectrolyte complex-interpenetrating network [PEC-IPN], poly­(dihydroxyphenyl-alanine-*co*-3-[methacryloylamino]­propyltrimethylammonium chloride) [P­(DOPA-MPTC)], chitosan quaternary ammonium salt [HACC], sodium hyaluronate [HA], 2-acrylamido-2-methylpropane sulfonic acid [AMPS], (2-[acryloyloxy]­ethyl)­trimethylammonium chloride [AETAC], (2-[methacryloyloxy]­ethyl)­dimethyl-(3-sulfopropyl)­ammonium hydroxide [MEDSAH], poly­(diallyldimethylammonium chloride) [PDADMAC], hyaluronic acid methacrylate [HAMA], [3-(methacryloylamino)­propyl]­trimethylammonium chloride [MPTC], 2-(dimethylamino)­ethyl methacrylate [DMAEMA], sodium *p*-styrenesulfonate [NaSS], methyl chloride quaternized *N*,*N*-dimethylamino ethacrylate [DMAEA-Q], and double network [DN]. Note: [+], [−], and [±] denote cationic, anionic, and zwitterionic charges, respectively.

As noted above, covalent cross-linking may be synergistically combined with electrostatic interactions to form ‘covalent-PEC hybrid’ hydrogels. For instance, reversible Schiff base cross-linking was utilized by Lv et al. to prepare antibacterial hydrogels with injectability, self-healing, and wet-adhesion.^61^ In other scenarios, irreversible covalent cross-linking may be introduced, particularly toward achieving mechanical robustness. For instance, Li et al., created PEC-IPN hydrogels, comprised of a covalent PEO-network surrounded by a PEC by employing cationic and anionic triblock PEs and photo-cross-linkable PEO-diacrylate (PEO–DA).^62^ In Lu et al., PEC-IPN hydrogels were prepared by the application of blue light to a mixture of zwitterionic poly­(dihydroxyphenyl-alanine-*co*-3-[methacryloylamino]­propyltrimethylammonium chloride) [P­(DOPA-MPTC)] and allyl-functionalized cellulose ether, inducing cross-linking of the latter.^63^ In Chen et al., covalently cross-linked PEC hydrogels were formed via “kneading-annealing” of cationic chitosan quaternary ammonium salt (HACC) and anionic sodium hyaluronate (HA), along with a photoinitiator and cross-linker.^64^ Wang et al. prepared covalently cross-linked PEC hydrogels with exceptional extensibility (strain ∼ 900%) and as well as adhesivity via free radical polymerization of acrylated zwitterionic MEDSAH with either cationic (2-acryloyloxy)­ethyl]­trimethylammonium chloride solution (AETAC) or anionic 2-acrylamido-2-methylpropanesulfonic acid (AMPS).^65^ Iudin et al. reported that exposure of 3D printed, covalently cross-linked hydrogels based on anionic methacrylated hyaluronic acid (HAMA) to a solution of cationic poly­(diallyldimethylammonium chloride) (PDADMAC) induced tunable shrinkage.^66^ In Zhang et al., a covalently cross-linked PA hydrogel with pH responsive shape memory was prepared via copolymerization of cationic 2-(dimethylamino)­ethyl methacrylate) (DMAEMA) and 3-(methacryloylamino)­propyl]­trimethylammonium chloride) (MPTC) with anionic sodium *p*-styrenesulfonate (NaSS) in the presence of a cross-linker.^67^ In Shao et al., salt responsive PA hydrogels were synthesized via copolymerization of anionic NaSS and cationic methyl chloride quaternized *N*,*N*-dimethylamino ethacrylate (DMAEA-Q) in the presence of a cross-linker.^68^ In Martinez et al., we developed a new hydrogel carrier system [‘cargo-carrying adhesive biosensor (CABs)’], comprised of a hollow rod and caps to house emerging optical glucose sensing assays.^69^ A CAB was readily assembled by contacting the rod with anionic surface charge and semi-IPN PA caps together with adhesivity achieved via electrostatic attraction between their opposite charges. Dynamic cross-linking afforded by electrostatic interactions expands the future development of biomedical hydrogels with tailored characteristics (e.g., adhesion, controlled release, self-healing, and bacterial resistance).

## 3 Hydrophobic Interactions

Dynamic hydrophobic interactions arise in hydrogels from amphiphilic polymers that bear hydrophobic and hydrophilic constituents.^70,71^ In an aqueous environment, amphiphilic polymers are thermodynamically driven to self-assemble into micellar-like structures so as to reduce hydrophobic contact with water.^72,73^ When hydrophobic and hydrophilic monomers are combined in an aqueous solution with a surfactant, micellular copolymerization may occur to localize the hydrophobic portion in the core. Hydrophobic micelles serve as dynamic cross-links, and are known to impart hydrogels with self-healing behavior and mechanical toughness, as well as reduced aqueous swelling.^74^ Additionally, inverse thermal responsive behavior is typically observed for hydrogels having hydrophobic interactions,^75^ with cyclical deswelling and reswelling occurring at temperatures above and below the volume phase transition temperature (VPTT), respectively. This has prompted their use in controlled drug delivery.^76–79^ Such thermally responsive hydrogels are formed from amphiphilic polymers that exhibit lower critical solution temperatures (LCST) in water. A notable example for biomedical applications is poly­(*N*-isopropylacrylamide) (PNIPAAm), which displays hydrophilic acrylamide and hydrophobic isopropyl moieties, and a LCST near physiological temperatures (∼32 °C) (Figure 4). Other polymers with similar LCSTs include poly­(*N*-vinylcaprolactam) [PNVCL] (LCST: 30–34 °C),^80–83^ poly­(vinyl methyl ether) [PMVE] (LCST: ∼30–40 °C),^84–86^ poly (*N*,*N*-diethylacrylamide) [PDEAAm] (LCST: ∼33 °C),^87–89^ and poly­([2-dimethylamino]­ethyl methacrylate) [PDMAEMA] (LCST: 40–50 °C)^90–93^ (Figure 5). Incorporation of hydrophilic or hydrophobic comonomers or blocks can be used to strategically increase or decrease, respectively, the LCST of polymers including PNIPAAm.^93–99^ The LCST of poly­(ethylene glycol) (PEG) (∼80–100 °C)^93,100^ has also been reduced by incorporation of hydrophobic moieties.^101,102^

**Figure 4:**
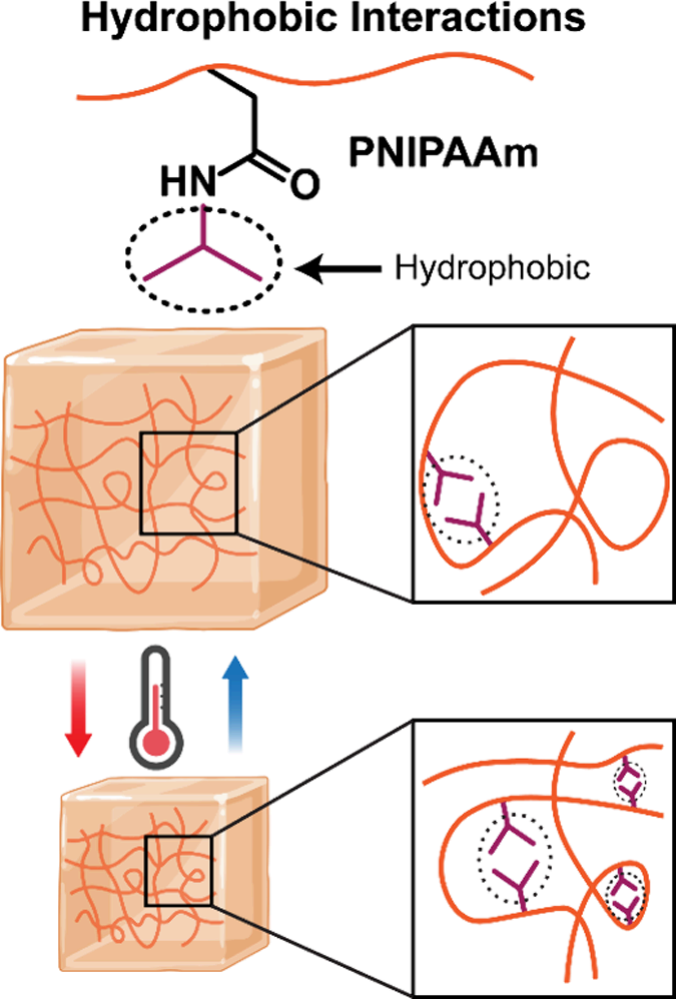
PNIPAAm hydrophobic interactions actuated by the temperature.

**Figure 5:**
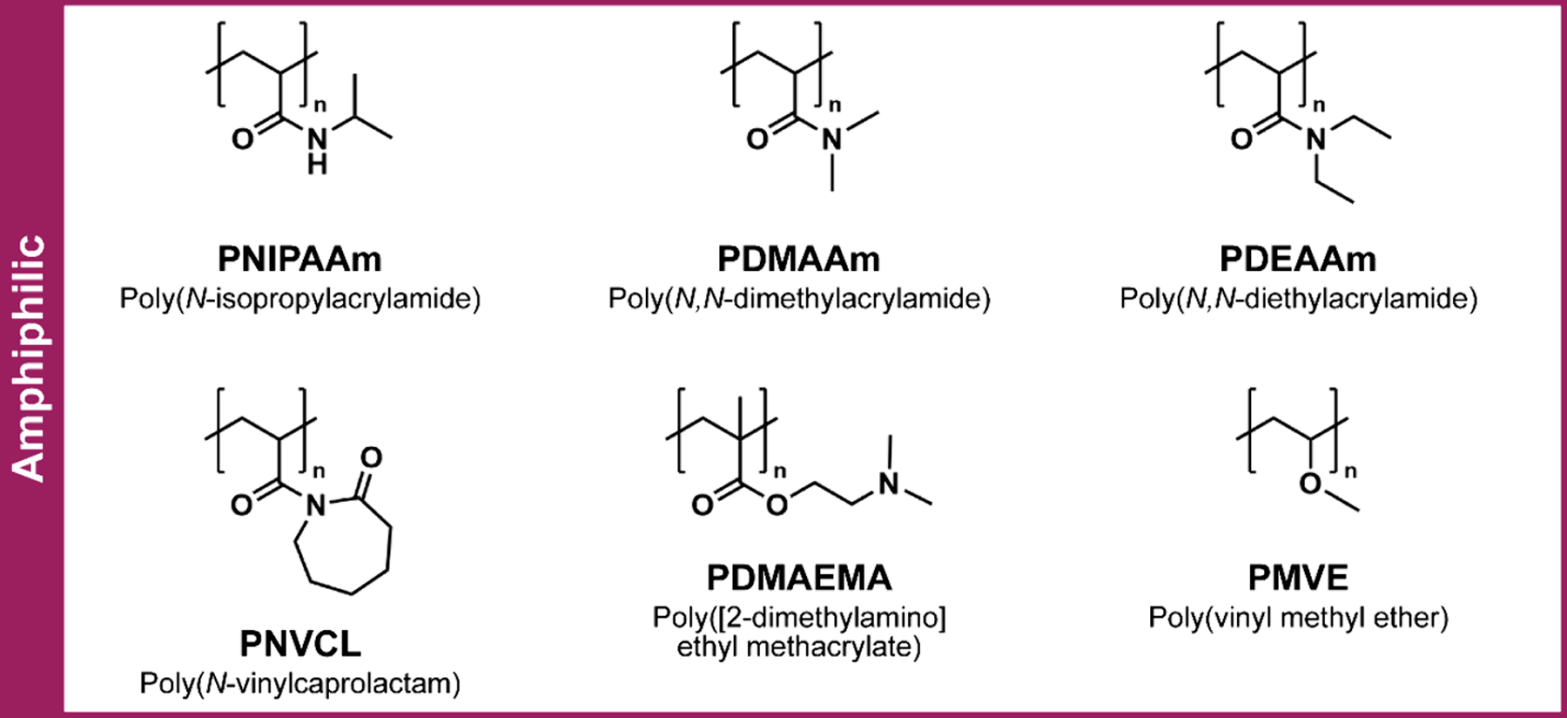
Commonly used amphiphilic polymers to form dynamic biomedical hydrogels via hydrophobic interactions.

Recent reports highlight the utility of hydrophobic interactions to produce hydrogels with distinct properties (Table 2). Zhou et al. prepared injectable, self-healing hydrogels for hydrophobic drug delivery based on combining octa-cyclodextrin polyhedral oligomeric silsequioxane (OCDPOSS) and acrylamide-modified adamantane (Ad-AAm).^103^ In Podda et al., polyacrylamide (PAAm) and polyoctadecylacrylate (C18A) were combined with divinylbenzene (DVB) to produce self-healing hydrogels with thermosensitive shape memory.^104^ Vahdati et al. reported an injectable, adhesive hydrogel prepared from PNIPAAm-*graft*-poly­(*N,N*-dimethylacrylamide) (PDMAAm) (PNIPAAm-*g*-PDMAAm) that exhibited strain hardening.^105^ Alternatively, Gou et al. conjugated PNIPAAm to hyaluronan (HA) via a PEG-based linker, and resulting in an injectable, hydrogel for subdermal delivery and sustained release.^106^ Zhang et al. produced hydrogels using poly­(poly­(*N*-alkyl-*N*-vinylacetamide) (PNANVA), having 3 mol % of alkyl side chains, resulting in increased strength for longer alkyl side chains.^107^

**Table 2:** Recent Studies of Hydrogels That Leverage Dynamic Hydrophobic Interactions^a^

	study	composition	key findings
**physically cross-linked hydrogels**	Zhou, 2020 ^103^	OCDPOSS/Ad-AAm	Injectable, self-healing hydrogels for hydrophobic drug delivery.
	Podda, 2023 ^104^	PAAm/C18A/DVB	Self-healing hydrogels with thermally driven shape memory behavior.
	Vahdati, 2020 ^105^	PNIPAAm-*g*-PDMAAm and PDMAAm-*g*-PNIPAAm	Both copolymers formed injectable, adhesive hydrogels, with PNIPAAm-*g*-PDMAAm exhibiting strain hardening.
	Gou, 2023 ^106^	HA-L-PNIPAAm	Injectable hydrogel for subdermal delivery (i.e., gelation at body temperature) and sustained release.
	Zhang, 2023 ^107^	PNANVA	Reversible hydrogel formation driven by alkyl side chains
	An, 2023 ^108^	hydrophilic monomers: AA, gelatin	Wet adhesion, with low swelling ratio resistant to buffered solutions to maintain adhesion.
		hydrophobic cross-linker: EDMA	
**covalently cross-linked hydrogels**	Tan, 2022 ^109^	hydrophilic monomer: AAm	Dynamic hydrophobic dissipated energy for extreme cyclic deformation (10,200% strain).
		hydrophobic monomer: DVB	
	He, 2022 ^110^	hydrophilic monomers: HEAA	Hydrogels formed with MXene conductive nanosheets were skin adhesive, and strain sensitive.
		hydrophobic monomer: PEA, MEA	
	Chen, 2022 ^111^	amphiphilic monomer: HPA	Covalently cross-linked hydrogels with wide thermoresponsive temperature range.
	Suljovrujic, 2023 ^112^	PEG-MA/PPG-MA	Covalently cross-linked hydrogels with physiological thermoresponsive behavior and noncytotoxic.

a Abbreviations: Octa-cyclodextrin polyhedral oligomeric silsequioxane [OCDPOSS], acrylamide-modified adamantane [Ad-AAm], polyacrylamide [PAAm], polyoctadecylacrylate [C18A], divinylbenzene [DVB], poly­(*N*-isopropyl acrylamide)-*graft*-poly­(*N*,*N*-dimethylacrylamide) [PNIPAAm-*g*-PDMAAm], linear hyaluronic acid-cyclcoctyne linker-poly­(*N*-isopropylacrylamide) [HA-L-PNIPAAm], poly­(*N*-alkyl-*N*-vinylacetamide) [PNANVA], acrylic acid [AA], ethylene dimethacrylate [EDMA], acrylamide [AAm], *N*-(2-hydroxyethyl)­acrylamide [HEAA], 2-phenoxyethyl acrylate [PEA], 2-methoxyethyl acrylate [MEA], 2-hydroxypropyl acrylate [HPA], poly­(ethylene glycol) methacrylate [PEG-MA], and poly­(propylene glycol) methacrylate [PPG-MA].

Covalent cross-links may also be introduced to hydrogels with hydrophobic interactions, typically for the purpose of enhancing mechanical properties. For instance, An et al. reported *in situ* polymerization of gelatin, and acrylic acid with a hydrophobic cross-linker (ethylene dimethacrylate) to form cross-linked hydrogels with antihydration (i.e., sustained wet tissue adhesion) and toughness.^108^ In Tan et al., mechanically robust (e.g., self-recoverable ultrahigh stretchability) hydrogels were produced by *in situ* polymerization of acrylamide (AAm) in the presence of divinyl benzene (DVB) cross-linker and a surfactant.^109^ He et al. copolymerized 2-phenoxyethyl acrylate (PEA), 2-methoxyethyl acrylate (MEA), and *N*-(2-hydroxyethyl) acrylamide (HEAA) in the presence of a cross-linker and MXene conductive nanosheets to form skin adhesive, strain sensitive hydrogels.^110^ Chen et al., covalently cross-linked 2-hydroylpropyl acrylate (HPA) with modified concentration, cross-linker and solvents to create a wide temperature range (6–40 °C) of thermoresponsive hydrogels.^111^ In Suljovrujic et al., covalently cross-linked oligomers poly­(ethylene glycol) methacrylate (PEG-MA) and poly­(propylene glycol) methacrylate (PPG-MA) exhibited physiological thermoresponsive behavior and were noncytotoxic.^112^ The development of biomedical hydrogels leveraging dynamic hydrophobic interactions has far reaching utility to expand polymer–polymer interactions for a variety of tailored characteristics (e.g., injectability, self-healing, adhesion, thermosensitivity, and controlled release).

## 4 Synergistic Combination of Dynamic Electrostatic and Hydrophobic Interactions

The aforementioned reports highlight the utility of electrostatic or hydrophobic interactions to form functional hydrogels with unique properties. Additionally, these two types of dynamic interactions may be synergistically combined, and reports have recently emerged to highlight the potential of this approach for producing hydrogels in a variety of biomedical applications (Figure 6, Table 3).

**Figure 6:**
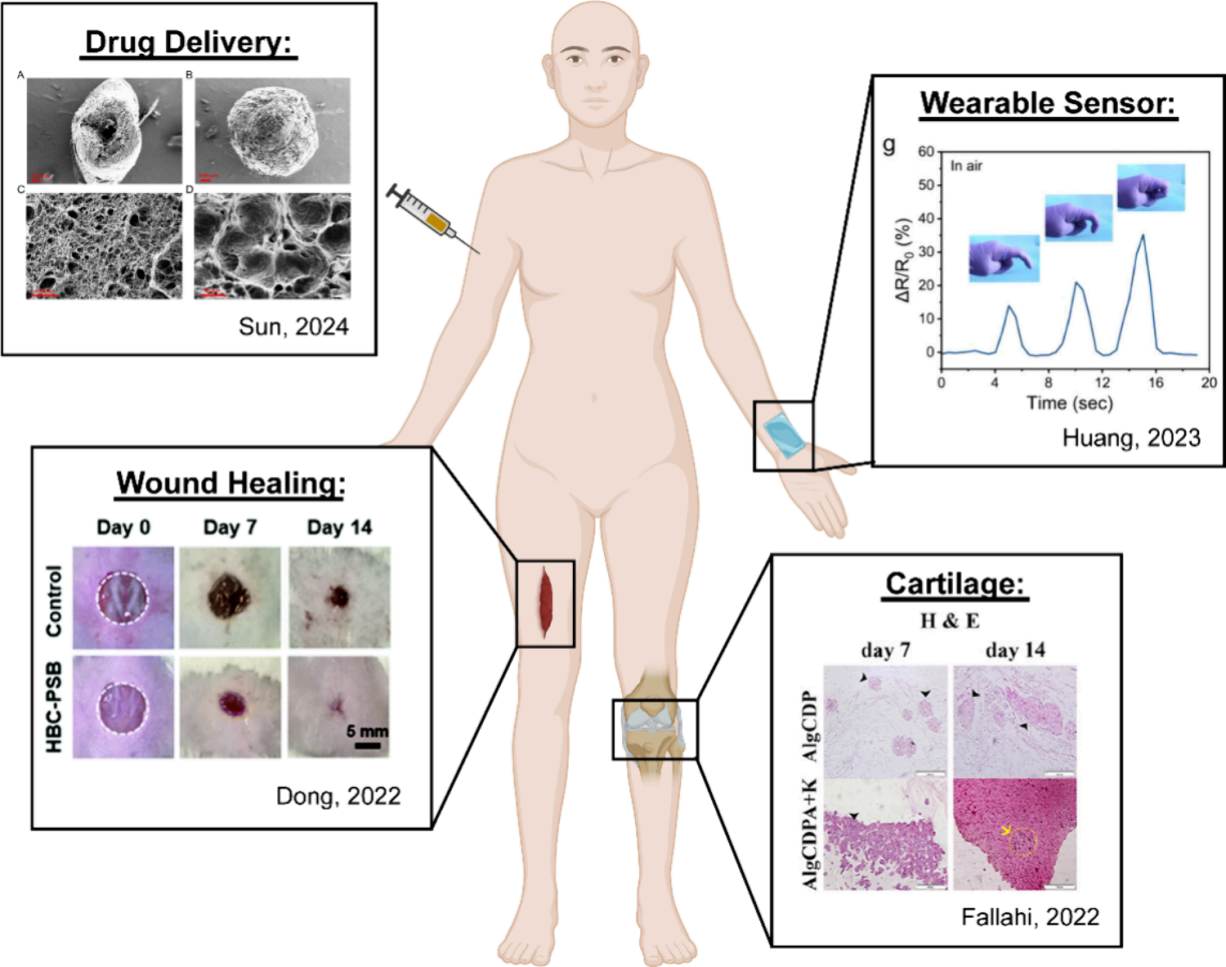
Applications of hydrogels prepared with both electrostatic and hydrophobic interactions.

**Table 3:** Recent Studies on Hydrogels That Leverage Combined Dynamic Electrostatic and Hydrophobic Interactions for Targeted Biomedical Applications^a^

	study	composition	key findings
**drug delivery**	Shafi, 2025 ^113^	electrostatic: CS [+], SDS [−]	Hydrogels exhibited gelation at body temperature, stability in the presence of NaCl at its concentration isotonic with blood, and controlled release.
		hydrophobic: SDS, Pluronic	
	Sun, 2024 ^114^	electrostatic: CS [+], tripolyphosphate [−], phospholipid head [−]	NLC hydrogel particles loaded with hydrophobic drugs exhibited improved shelf-stability and drug release.
		hydrophobic: phospholipid tail	
	Hoang, 2022 ^115^	electrostatics: COS-Nb [+], Alg-Tz [−]	Dual cross-linked hydrogel (electrostatics and click) responsive to pH to release drugs at higher pH (intestinal tract) with improved strength and thermal stability.
		hydrophobic: polysaccharide	
**articular cartilage**	Fallahi, 2022 ^116^	electrostatics: P-A [+], Alg [−]	Supramolecular injectable hydrogel with improved shear modulus and extended *in vitro* hydrogel degradation.
		hydrophobic: Pluronic, β-CD	
	Demott, 2023 ^117^	electrostatic: AMPS [−], APTAC [+]	Electrostatic attraction and hydrophobic associations imparted transitional cartilage mimetic stiffness, strength, and hydration.
		hydrophobic: NIPAAm (isopropyl)	
	Dingus, 2025 ^118^	electrostatic: AMPS [−], APTAC [+]	Polyampholyte 3rd network imparted increased adhesion to create a partial thickness chondral defect replacement hydrogel.
		hydrophobic: NIPAAm (isopropyl)	
**wound healing**	Dong, 2022 ^119^	electrostatic: PSBMA [±], HBCS [+]	Thermoresponsive, injectable hydrogel with rapid self-healing and antibacterial behavior.
		hydrophobic: HBCS [+]	
	Durand, 2025 ^120^	electostatic: CS [+], DOTAGA-CS [±]	Saline and pH responsive injectable hydrogel with rapid self-assembly and stability.
		hydrophobic: CS [+]	
	Li, 2021 ^121^	electrostatic: PAA–[VBIm]Br [+], MGA [−]	Hydrophobic interactions improved water absorption and ionic liquid promoted self-healing and antibacterial activity.
		hydrophobic: MGA	
**wearable sensors**	Huang, 2023 ^122^	electrostatic: PAETAC [+], STPP [−]	Increased conductivity, strain, and adhesion of hydrophobic hydrogels for flexible strain sensors.
		hydrophobic: PEA	
	Li, 2025 ^123^	electrostatic: PAA [−], LM (Ga^3+^, In^3+^) [+]	Imparted high strain recovery and self-healing of flexible touch panel and strain sensor.
		hydrophobic: DVB, SDS	
	Wang, 2023 ^124^	electrostatic: Zn^2+^ [+], Mn^2+^ [+], SO_4_^2–^ [−]	Improved dispersion to inhibit Zn dendrite, with high self-healing efficiency.
		hydrophobic: OMA (alkyl)	
	Fu, 2023 ^125^	electrostatic: PTA [−], SDS [−], Fe^3+^ [+]	Pressure-sensitive adhesive (PSA) with increased adhesion imparted by removal of interfacial water.
		hydrophobic: OMA (alkyl)	

a Abbreviations: Chitosan [CS], sodium dodecyl sulfate [SDS], poly­(ethylene oxide)-*block*-poly­(propylene oxide)-*block*-poly­(ethylene oxide) [Pluronic], nanostructured lipid carriers [NLC], norbornene-fuctionalized chitosan oligosaccharide [COS-Nb], tetrazine-functionalized aliginate [Alg-Tz], pluronic-amine [P-A], alginate [Alg], β-cyclodextrin [β-CD], 2-acrylamido-2-methylpropanesulfonic acid [AMPS], (3-acrylamidopropyl)­trimethylammonium chloride [APTAC], *N*-isopropylacrylamide [NIPAAm], poly­(sulfobetaine methacrylate) [PSBMA], hydroxylbutyl chitosan [HBCS], 1,4,7,10-tetraazacyclododecane-1-glutaric acid-4,7,10-triacetic acid functionalized chitosan [DOTAGA-CS], 1-vinyl-3-butylimidazolium modified poly­(acrylic acid) [PAA-[VBIm]­Br^**+**^], modified gum arabic [MGA], poly­([2-(acryloyloxy)­ethyl]­trimethy-lammonium chloride) [PAETAC], sodium phosphate solutions [STPP], poly­(ethyl acrylate) [PEA], poly­(acrylic acid) [PAA], liquid metal [LM], divinylbenzene [DVB], octadecyl methacrylate [OMA], and phosphotngstic acid [PTA]. Note: [+], [−], and [±] denote cationic, anionic, and zwitterionic charges, respectively.

### Drug Delivery

An array of hydrophobic drugs (e.g., corticosteroids, nonsteroidal anti-inflammatory drugs [NSAIDs], hormones, and chemotherapeutics) are used to treat a variety of conditions.^126^ Adequate loading and release by hydrogels, as well as stability in various physiological environments associated with a delivery pathway (e.g., intravenous, transdermal, and gastrointestinal [GI] tract), presents challenges.^126^ The combination of electrostatic interactions and hydrophobic interactions to achieve hydrogels with such behavior has recently been recently demonstrated. In Shafi et al., cationic chitosan (CS) was combined with an anionic, amphiphilic sodium dodecyl sulfate (SDS) and amphiphilic Pluronic (i.e., PEO-*block*-PPO-*block*-PEO) to afford sustained release of a hydrophobic drug.^113^ Gelation was not compromised in the presence of isotonic NaCl; however, when SDS was excluded, gelation was diminished due to the resulting charge shielding of CS cations by chloride anions and reduced hydrophobic interactions. PEC hydrogels have been prepared by combining cationic CS with anionic tripolyphosphate (TPP).^127^ However, the addition of hydrophobic interactions may afford a superior drug delivery of hydrophobic drugs. For instance, Sun et al. prepared chitosan–lipid nanocarriers (NLC–CS) by combining CS and TPP with a phospholipid having an anionic headgroup and hydrophobic tail.^114^ When loaded with a hydrophobic drug, these NLC–CS particles achieved excellent shelf stability and skin permeation. Hoang et al. prepared pH responsive hydrogels based on cationic norbornene-functionalized CS oligosaccharide (COS-Nb) and anionic tetrazine-functionalized alginate (Alg-Tz).^115^ The click reaction between Nb and Tz groups afforded chemical cross-links that were also capable of hydrophobic interactions. This cross-linking strategy afforded hydrogels that were stable in acidic environments such as the GI tract.

### Articular Cartilage

Damaged articular cartilage requires surgical intervention, as a result of avascularity and subsequent low healing capacity.^128,129^ Hydrogels have been extensively studied to restore cartilage tissue, including scaffolds for regeneration.^130,131^ A hydrogel scaffold that employs both electrostatic and hydrophobic interactions has an interesting potential for cartilage regeneration. In Fallahi et al., anionic β-cyclodextrin-grafted alginate (β-CD-Alg) and cationic Pluronic-amine (i.e., PEO-*block*-PPO-*block*-PEO amine; P-A) were combined to form an injectable hydrogel to deliver mesenchymal stem cells (MSCs) and pro-chondrogenic growth factors to cartilage lesions.^116^ In addition to electrostatic interactions, hydrophobic interactions occurred between the β-CD core and the Pluronic PPO blocks. Owing to the synergy of dynamic bonding, the resulting hydrogel possessed a compressive modulus (*E*_c_) (∼5.6 kPa) that was greater than that of alginate alone (∼3.2 kPa) or β-CD-Alg without Pluronic (∼3.9 kPa). Additionally, the β-CD core afforded loading and sustained release with kartogenin growth factor, facilitating chondrogenic differentiation of the encapsulated MSCs. In the case of articular cartilage substitutes, a hydrogel should immediately mimic mechanical properties as well as high hydration (70–90%) of native tissue,^132^ such as superficial cartilage (i.e., the topmost zone) [*E*_c_ ∼ 1 MPa and strength (σ_c_) ∼ 15 MPa] and transitional cartilage (i.e., the middle zone) [*E*_c_ ∼ 2–4 MPa and σ_c_ ∼ 20–40 MPa].^128,133–137^ In conjunction with a multinetwork design, hydrogels that combine electrostatic interactions and hydrophobic interactions represents a promising approach to achieve cartilage-like mechanical properties. Gong et al. reported an electrostatically repulsive double network (DN) hydrogel, prepared from an anionic poly­(2-acrylamido-2-methylpropanesulfonic acid) (PAMPS; 1st network; ‘highly’ covalently cross-linked), and a neutral polyacrylamide (PAAm; 2nd network; ‘lightly’ covalently cross-linked).^138^ This PAMPS/PAAm DN hydrogel exhibited a high water content (∼80%) and ultrahigh σ_c_ (∼17 MPa), but a sub-MPa *E*_c_ (∼300 kPa). In Means et al., we prepared DN hydrogels that also incorporated hydrophobic interactions by forming a second network based on amphiphilic *N*-isopropylacrylamide (NIPAAm).^139^ Since thermosensitivity is not desired for cartilage substitutes, NIPAAM was copolymerized with AAm to increase the VPTT above the physiological range. The resulting PAMPS/P­(NIPAAm-*co*-AAm) DN hydrogel had superficial articular cartilage-like properties, including an unprecedented combination of high hydration (∼85%), *E*_c_ ∼ 1 MPa, and σ_c_ ∼ 25 MPa. More recently, in Demott et al., we reported a triple network (TN) hydrogel formed by the addition of a cationic third network based on 3-(acrylamidopropyl)­trimethylammonium chloride (APTAC).^117^ For the resulting PAMPS/P­(NIPAAm-*co*-AAm)/PAPTAC TN hydrogels, electrostatic interactions formed between the anionic first network and the cationic third network, in addition to the hydrophobic interactions within the second network. As the concentration of the cationic third network was increased, the moduli increased before ultimately plateauing. In this way, a TN hydrogel exhibiting properties of transitional cartilage was achieved, with hydration (∼80%), *E*_c_ ∼ 3 MPa, and σ_c_ ∼ 30 MPa (i.e., greater *E*_c_ and σ_c_ versus the DN hydrogel lacking the cationic third network). This TN hydrogel was subsequently utilized to create a 2-layered hydrogel construct that recapitulated the superficial and transitional layers of articular cartilage.^118^ To create a superficial layer that was adhesive to the cationic surface of the TN hydrogel (i.e., transitional layer), an analogous TN hydrogel was formed but with a PA third network consisting of 30:70 ratio of cationic APTAC and anionic AMPS.^118^

### Wound Healing

Hydrogels wound dressings that offer increased efficacy (e.g., injectability and antimicrobial) holds potential to treat a variety of thermal and physical wounds, as well as wound ulcers.^140,141^ Examples of hydrogel wound dressings that combine electrostatic and hydrophobic interactions have recently emerged. For instance, Dong et al. prepared a hydrogel wound dressing by combining polyzwitterionic poly­(sulfobetaine methacrylate) (PSBMA) and cationic, amphiphilic hydroxybutyl CS (HBCS).^119^ Electrostatic interactions arose from oppositely charged groups, and the hydroxybutyl (HB) groups imparted hydrophobic interactions. Additionally, covalently cross-linking was afforded by the UV cure of the methacrylate groups of PSBMA. The resulting thermoresponsive hydrogel was injectable, capable of gelation at body temperature, self-healing, antibacterial, and demonstrated capacity to heal infected wounds. In Durand et al., cationic CS was functionalized with an anionic polycarboxylic macrocyle (1,4,7,10-tetraazacyclododecane-1-glutaric acid-4,7,10-triacetic acid; DOTAGA).^120^ Hydrophobic interactions arose from the hydrophobic moieties of the DOTAGA. The resulting injectable hydrogel exhibited gelation under physiological conditions as well as degradation *in vivo*. In Li et al., poly­(acrylic acid) (PAA) was modified with 1-vinyl-3-butylimidazolium ([VBIm]­Br^+^), and the resulting PAA–[VBIm]­Br^+^ was then combined with carboxylic acid modified gum arabic and aluminum chloride.^121^ A hydrogel was formed by the electrostatic interactions between PAA–[VBIm]­Br^+^ and Al^3+^ with carboxylic acid anions (−COO^–^) of gum arabic as well as hydrophobic interactions imparted by the amphiphilic gum arabic. This hydrogel was antibacterial, self-healing, and demonstrated accelerated healing of a full-thickness skin defect.

### Wearable Sensors

Hydrogels have been utilized in numerous wearable sensors to detect and distinguish various stimuli (e.g., mechanical, thermal, and bioelectrical).^142–144^ Hydrogels with both electrostatic and hydrophobic interactions that impart properties critical to wearable sensors. For instance, Huang et al. immersed a cationic poly­(ethyl acrylate-*co*-2-(acryloyloxy)­ethyl trimethylammonium chloride) p­(EA-*co*-DAC) hydrogel into anionic sodium phosphate solutions (STPP), resulting in electrostatic interactions.^122^ Hydrophobic interactions also occurred among the ethyl moieties. These hydrogels achieve high toughness, low hysteresis, strain sensitivity, and fast self-recovery. Li et al. reported a conductive hydrogel prepared by exposure of an aqueous dispersion of acrylic acid (AA) monomer, sodium dodecyl sulfate (SDS) surfactant, anionic divinylbenzene (DVB) cross-linker, and liquid metal (LM) to γ-radiation.^123^ This resulted in ‘hydrophobic nodes’ capable of hydrophobic interactions, and electrostatic interactions occurred between anionic carboxylic acid moieties (−COO−) of PAA and metallic cations of the LM (Ga^3+^ and In^3+^). This PAA-DVB-LM hydrogel exhibited remarkable stretchability (∼5000% elongation at break in tension), self-healing, and excellent responsiveness as a strain sensor. In Wang et al., hydrophobic octadecyl methacrylate (OMA) was copolymerized with hydrophilic acrylamide (AAm) in the presence of a covalent cross-linker (*N*′,*N*′-methylenebis­(acrylamide), BIS) in an aqueous solution containing SDS and initiator, as well as ZnSO_4_ and MnSO_4_.^124^ Following free-radical polymerization, the resulting hydrogel electrolyte exhibited electrostatic interactions as well as hydrophobic interactions of the OMA segments and displayed self-healing and high ionic conductivity. It was also used to form Zn/MnO_2_ batteries that displayed high specific capacity and durability. A number of current wearable sensors (e.g., continuous glucose monitors [CGMs], drug delivery patches, and ECG patches) require a robust pressure sensitive adhesive for secure skin fixation.^145^ Toward this goal, Fu et al. developed pressure-sensitive adhesive hydrogels via the copolymerization of AAm and OMA in the presence of anionic phosphotungstic acid (PTA), a type of metal oxide nanocluster.^125^ Subsequent treatment with Fe^3+^ afforded electrostatic interactions with PTA. Hydrophobic interactions stemmed from the association of the OMA moieties. This P­(AAm-*co*-OMA)/PTA-Fe hydrogel produced robust dry and wet adhesivity, including saline solutions.

## 5 Conclusions

Hydrogels that possess dynamic interactions (i.e., cross-linking) have the potential to access distinct properties versus traditional hydrogels prepared with irreversible covalent cross-links. Such hydrogels have attracted significant interest in biomedical applications, as they are characterized by greater responsiveness to external stimuli encountered in the body and robust mechanical properties such as self-healing as well as tissue adhesivity. These properties have prompted their exploration in drug delivery, tissue repair and replacement, wound dressing, and sensing. A variety of dynamic covalent and physical interactions exist. However, as highlighted herein, electrostatic and hydrophobic interactions have demonstrated particular efficacy to form functional hydrogels. While fewer in example, hydrogels created with both electrostatic and hydrophobic interactions hold promise to access further improved properties. To produce electrostatic interactions, a variety of PE and PA chemistries are available, while numerous amphiphilic polymers can give rise to hydrophobic associations. These interactions can be tuned and magnified with polymer architectures and network structures (e.g., IPNs, semi-IPNs). Additionally, covalent cross-linking can be introduced to further tailor properties. Advancing the utility of dynamic hydrogels will rely on robust characterization, including *in vitro* testing that recapitulates physiological environments. Furthermore, testing should be done with standard processes, including degradation (ASTM F1635–24),^146^ mechanical (ASTM D638–22, ASTM F732–17, F2150–19),^147–149^ and cytotoxicity (ISO 10993–5).^150^ Promising systems should also be demonstrated *in vivo* using appropriate standards (ISO 10993)^151^ and preclinical models. Overall, hydrogels that leverage dynamic interactions, especially electrostatic and hydrophobic interactions, have great potential to fulfill the requirements necessary in numerous biomedical applications.
